# Seamless QM/MM Simulations via a GROMACS-CP2K Interface

**DOI:** 10.1021/acs.jcim.6c02073

**Published:** 2026-09-10

**Authors:** Dmitry Morozov, Christian Blau, Ole Schütt, Vladimir Mironov, Arno Proeme, Holly Judge, Thomas D. Kühne, Berk Hess, Gerrit Groenhof

**Affiliations:** † Nanoscience Center and Department of Chemistry, 4168 University of Jyväskylä, P.O. Box 35, Jyväskylä FI-40014, Finland; ‡ Department of Applied Physics, Science for Life Laboratory, KTH Royal Institute of Technology, Solna SE-17165, Sweden; § CP2K Foundation, Zürich CH-8057, Switzerland; ∥ 702880 Terra Quantum AG, Kornhausstrasse 25, St. Gallen CH-9000, Switzerland; ⊥ 601204 EPCC, The University of Edinburgh, Bayes Centre, 47 Potterrow, Edinburgh EH8 9BT, U.K.; # 682670 Center for Advanced Systems Understanding (CASUS), Untermarkt 20, Görlitz 02826, Germany; ∇ Helmholtz-Zentrum Dresden-Rossendorf (HZDR), Bautzner Landstraße 400, Dresden 01328, Germany; ○ Institute of Artificial Intelligence, Technische Universität Dresden, Helmholtzstraße 10, Dresden 01069, Germany

## Abstract

Understanding and controlling chemical reactivity in biological systems require atomic-level insight into processes that are often inaccessible to experiments. Hybrid quantum mechanics/molecular mechanics (QM/MM) simulations provide a powerful framework for describing chemical reactions in complex environments, but their practical application remains limited by fragmented software ecosystems, restricted accessibility, and methodological approximations that can compromise accuracy and reproducibility. In particular, many existing QM/MM implementations rely on ad hoc couplings, proprietary software, or truncated treatments of long-range electrostatic interactions. Here, we present a robust and fully periodic QM/MM interface between the open-source molecular dynamics engine GROMACS and the electronic structure theory code CP2K. The implementation enables efficient and reproducible QM/MM molecular dynamics and enhanced sampling simulations with a consistent treatment of long-range electrostatics under periodic boundary conditions. By combining the strengths of two widely used community codes, this interface provides a general and scalable platform for studying chemical reactivity in biological systems and establishes a transparent reference implementation for QM/MM simulations.

## Graphical abstract

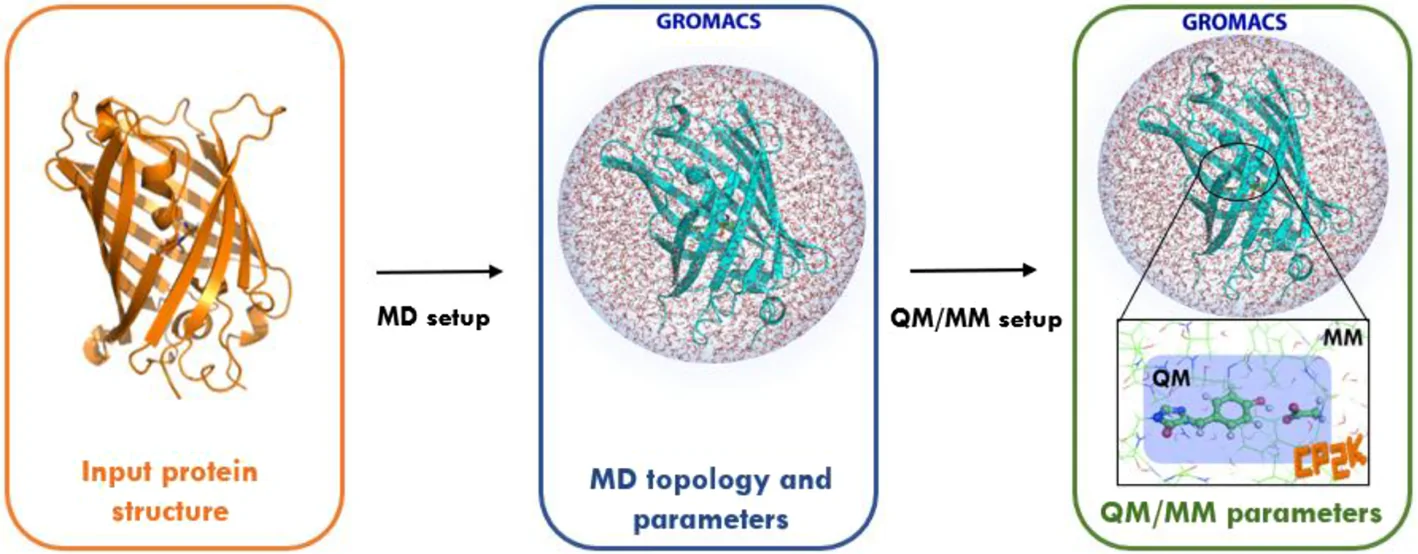

## 1 Introduction

Understanding how enzymes mediate chemical reactions is central to the development of new and efficient catalysts. Because the spatial and temporal resolution required to obtain atomistic insight into enzymatic mechanisms is difficult to achieve experimentally, computer simulations have become essential tools for studying enzymatic chemistry. In particular, hybrid quantum-mechanical/molecular-mechanical (QM/MM) simulations are widely used to investigate reaction mechanisms in complex biological environments.^1,2^ Despite major methodological and software advances since the introduction of QM/MM approaches almost five decades ago,^3^ reliable biomolecular QM/MM calculations still require careful choices concerning the QM/MM partition, electrostatic coupling, electronic structure level, and sampling strategy, as well as trade-offs between accuracy and computational costs.^4–6^

First, the reliability of QM/MM simulations depends critically on the accuracy of the underlying QM/MM potential,^7^ which is determined not only by the electronic structure method used for the quantum region and the force field describing the molecular mechanics region but also by the manner in which these two subsystems are coupled. Accurate treatment of long-range electrostatic interactions is particularly important in biomolecular systems, where electrostatic effects can strongly influence the reaction energetics. Second, enzymatic reactions typically occur on time scales that are far beyond those accessible to unbiased QM/MM molecular dynamics simulations, which are often limited to a few picoseconds at practical levels of theory. Although a variety of enhanced sampling techniques have been developed to address this limitation,^8^ their integration with QM/MM simulations remains technically demanding and often requires carefully chosen collective variables and substantial user expertise. Third, QM/MM implementations are often tightly coupled to specific software ecosystems, making it difficult to install, extend, maintain, or combine with modern sampling methodologies.

To address these challenges, we present a new interface between the molecular dynamics package GROMACS^9,10^ and the electronic structure theory code CP2K,^11,12^ enabling fully periodic QM/MM simulations within an open-source, high-performance-computing framework. The resulting QM/MM potential provides a consistent treatment of long-range electrostatic interactions under periodic boundary conditions (PBCs), thereby avoiding artifacts associated with electrostatic truncation. Combined with the accelerated weight histogram (AWH) enhanced-sampling method available in GROMACS,^13^ the interface enables an efficient and reproducible exploration of enzymatic reaction pathways. This work establishes a general, open, and scalable QM/MM framework designed to support a broad range of studies of chemical reactivity in biological systems.

## 2 Methods

The interface uses an additive QM/MM scheme for energies and forces ([Disp-formula eq1]):

1 $$\begin{array}{ll} E^{\mathrm{QM}/\mathrm{MM}} & =E^{CP2K}+E^{\mathrm{GROMACS}}\\ E^{CP2K} & =E_{\mathrm{QM}}+E_{\mathrm{QM}-\mathrm{MM}}^{\mathrm{CP}2K}\\ E^{\mathrm{GROMACS}} & =E_{\mathrm{MM}}+E_{\mathrm{QM}-\mathrm{MM}}^{\mathrm{LJ}}+E_{\mathrm{boundary}}^{\mathrm{bonded}}\\ F_{i}^{\mathrm{QM}/\mathrm{MM}} & =F_{i}^{\mathrm{CP}2K}+F_{i}^{\mathrm{GROMACS}}\\ F_{i}^{\mathrm{CP}2K} & =-\nabla_{i}E^{\mathrm{CP}2K}\\ F_{i}^{\mathrm{GROMACS}} & =-\nabla_{i}E^{\mathrm{GROMACS}} \end{array}$$

Here, *E*_QM_ is the energy of the QM subsystem and $E_{\mathrm{QM}-\mathrm{MM}}^{CP2K}$ is the electrostatic interaction with the MM environment, including polarization of the QM density by the MM charges. This is the physically important coupling for enzymatic reaction barriers: the environment polarizes the electronic density of the reacting region, while the extended protein, solvent, ions, and nucleic acids remain described by the force field.^3^ CP2K evaluates these contributions and the corresponding forces on both QM and MM atoms. The term *E*_MM_ contains force-field interactions involving only MM atoms. GROMACS additionally retains the Lennard-Jones interactions between QM and MM atoms, $E_{\mathrm{QM}-\mathrm{MM}}^{\mathrm{LJ}}$ , and the force-field bonded terms crossing the QM/MM boundary, $E_{\mathrm{boundary}}^{\mathrm{bonded}}$ . The interface uses link atoms to cap the broken bonds between subsystems. The CP2K and GROMACS force contributions are then summed before constraints and time integration are applied. Any restraint or enhanced-sampling bias potential and its forces are evaluated separately by GROMACS.

### 2.1 CP2K GPW Representation and Periodic QM/MM Energy

The CP2K part of the interface is not a black-box single-point calculator. It brings the Quickstep Gaussian and plane-wave (GPW) formulation into the GROMACS time-integration loop.^11,14,15^ In GPW, the Kohn–Sham orbitals are expanded ([Disp-formula eq2]) in localized Gaussian basis functions,

2 $$\psi_{i}\left(r\right)=\underset{\mu }{\sum }C_{\mu i}\phi_{\mu }\left(r\right)$$

whereas the electronic density and electrostatic potentials are represented on hierarchical real-space grids. The total QM charge density ([Disp-formula eq3]) used for electrostatics can be written schematically as

3 $$\rho_{\mathrm{QM}}\left(r\right)=\rho_{\mathrm{el}}\left(r\right)+\underset{A\in \mathrm{QM}}{\sum }Z_{A}g_{A}\left(r-R_{A}\right)$$

where ρ_el_ is the electronic density and *g*_*A*_ denotes the smooth Gaussian compensation charge associated with nucleus *A*. This mixed representation is central for periodic QM/MM because the chemically relevant density is retained in a localized Gaussian basis, while the expensive long-range electrostatic terms are evaluated on grids with fast Fourier transforms and multigrid interpolation.^16,17^

### 2.2 GEEP Electrostatics and Periodic Cell Decoupling

Under periodic boundary conditions, direct electrostatic coupling between a grid-based electronic density and bare MM point charges is numerically delicate. The singular point-charge potential can overpolarize the QM density when MM atoms approach the QM region, and a brute-force mapping of all MM Coulomb potentials to the finest CP2K grid would scale poorly for large biomolecular environments. CP2K resolves this through the fully periodic QM/MM formalism of Laino and coworkers and the Gaussian expansion of the electrostatic potential (GEEP).^18,19^

For an MM atom *a* with charge *q*_*a*_ at position **r**_*a*_, the bare electrostatic embedding contribution would involve

4 $$E_{\mathrm{QM}-\mathrm{MM},a}=q_{a}\int \frac{\rho_{\mathrm{QM}}\left(r\right)}{\left|r-r_{a}\right|}dr$$

In CP2K, this interaction is regularized by replacing the singular point-charge potential of ([Disp-formula eq4]) with a smooth, finite potential,

5 $$v_{a}^{\mathrm{smooth}}\left(r\right)=q_{a}\frac{\mathrm{erf}\left(\left|r-r_{a}\right|/R_{c,a}\right)}{\left|r-r_{a}\right|}$$

where *R*_*c,a*_ is an atom-dependent smoothing length, commonly related to the covalent radius. GEEP then rewrites this smooth potential as a short-range Gaussian expansion plus a slowly varying residual,

6 $$v_{a}^{\mathrm{GEEP}}\left(r\right)=q_{a}\left[\underset{\mathrm{fitted}}{\underset{︸}{\overset{N_{G}}{\underset{k=1}{\sum }}c_{ak}\exp\left(-\frac{{|r-r_{a}|}^{2}}{\sigma_{ak}^{2}}\right)}}+\underset{\mathrm{residual}}{\underset{︸}{R_{a}^{\mathrm{low}}\left(\left|r-r_{a}\right|\right)\;}}\right]$$

The Gaussian parameters *c*_*ak*_ and σ_*ak*_ are obtained in a least-square fitting procedure of the potential ([Disp-formula eq5]) in reciprocal space. The fitting procedure ensures that the Fourier components of the residual $R_{a}^{\mathrm{low}}$ are concentrated in the low-frequency domain.^18^ When CP2K’s internal GEEP library is used, the expansion length, *N*_G_, is selected through the CP2K input keyword USE_GEEP_LIB and is therefore common to all MM atoms within a calculation. The atom-kind-dependent smoothing radius, *R*_*c,a*_, rescales the corresponding Gaussian coefficients and widths. The automatically generated interface input uses *N*_G_ = 12; the values used for the individual test systems are reported in the Supporting Information. The Gaussian terms are placed on the coarsest CP2K multigrid level that can represent their width, while only sharp, short-ranged contributions reach the finer grids. The contribution of the smooth residual $R_{a}^{\mathrm{low}}$ introduced in ([Disp-formula eq6]) is always accumulated on the coarsest grid for all MM atoms. The resulting embedding energy is evaluated as

7 $$E_{\mathrm{QM}-\mathrm{MM}}^{CP2K}=\int \rho_{\mathrm{QM}}\left(r\right)\underset{a\in \mathrm{MM}}{\sum }v_{a}^{\mathrm{GEEP}}\left(r\right)dr$$

with forces on both QM and MM atoms obtained consistently from the same embedding potential. This is a central reason why the interface is more than a file-based driver: the expensive and delicate QM/MM electrostatics ([Disp-formula eq7]) remain inside CP2K, where GPW, GEEP, and the multigrid machinery are implemented and tested.

Periodic QM/MM introduces an additional subtlety. The full MM system may occupy a large periodic box, whereas the QM wave function should be represented in a compact CP2K cell whose size is determined by the reactive region. If the QM density were simply repeated with the small QM cell periodicity, the calculation would introduce artificial interactions between QM images. CP2K therefore decouples the QM density from its small-cell periodic replicas and recouples it to the periodicity of the full QM/MM simulation cell using the electrostatic decoupling strategy of Blöchl and related reciprocal-space treatments.^20,21^

The essential idea can be expressed as an electrostatic correction to the periodic Poisson problem. For a localized QM charge density ρ in the compact CP2K cell, the periodic electrostatic energy, *E* ^PBC^[ρ], contains interactions with periodic images. Blöchl’s construction introduces a model charge density, ρ̂, that is confined to the reference cell but reproduces the long-range electrostatic moments of ρ. A convenient representation is a sum of atom-centered normalized Gaussians ([Disp-formula eq8]):

8 $$\mathrm{\rho ̂}\left(r\right)=\underset{i}{\sum }\frac{q_{i}}{{\left(\sqrt{\pi }\sigma_{i}\right)}^{3}}\exp\left[-\frac{{|r-R_{i}|}^{2}}{\sigma_{i}^{2}}\right]$$

where the charges *q*_*i*_, centers **R**_*i*_, and widths σ_*i*_ are chosen so that ρ̂ matches the relevant low-order multipoles and the spatial extent of the true density. Because ρ–ρ̂ has a reduced long-range field, the isolated-cell (decoupled) electrostatic energy can be obtained by subtracting the periodic self-interaction of the model density from the energy of the periodic QM system ([Disp-formula eq9]). The model density ρ̂ is further used in a similar way ([Disp-formula eq10]) to introduce back the electrostatic interactions (recouple) between QM images within the full periodicity of the GROMACS (QM/MM) box:

9 $$\begin{array}{ll} E^{\mathrm{isolated}}\left[\rho \right] & \approx E_{\mathrm{QMcell}}^{\mathrm{PBC}}\left[\rho \right]-E_{\mathrm{QMcell}}^{\mathrm{coupl}}\left[\mathrm{\rho ̂}\right]\\ & =E_{\mathrm{QMcell}}^{\mathrm{PBC}}\left[\rho \right]-\left(E_{\mathrm{QMcell}}^{\mathrm{PBC}}\left[\mathrm{\rho ̂}\right]-E^{\mathrm{isolated}}\left[\mathrm{\rho ̂}\right]\right) \end{array}$$

10 $$\begin{array}{ll} E_{\mathrm{QMMMcell}}^{\mathrm{PBC}}\left[\rho \right] & \approx E^{\mathrm{isolated}}\left[\rho \right]+E_{\mathrm{QMMMcell}}^{\mathrm{coupl}}\left[\mathrm{\rho ̂}\right]\\ & =E^{\mathrm{isolated}}\left[\rho \right]+\left(E_{\mathrm{QMMMcell}}^{\mathrm{PBC}}\left[\mathrm{\rho ̂}\right]-E^{\mathrm{isolated}}\left[\mathrm{\rho ̂}\right]\right) \end{array}$$

This approach can also be used to decouple and recouple the periodic self-interaction contributions to the electronic Hamiltonian and the nuclear forces. Charged QM and MM partitions are handled with neutralizing background charge densities whose net contribution cancels in the total QM/MM energy.^19,20^ In practice, this separation of a compact QM cell from a large GROMACS simulation box is what makes fully periodic biomolecular QM/MM simulations feasible without sacrificing the long-range electrostatic environment.

### 2.3 Software Design and Integration of GROMACS with CP2K

The GROMACS side of the implementation is built as an external force provider using the unified MDModules API (Figure 1). The module receives the current coordinates and cell, identifies the QM and MM subsets, constructs the data needed by CP2K, and returns QM/MM energies and forces to the GROMACS integration loop. The core GROMACS tools remain agnostic to the details of the QM engines. This separation is important for maintainability because it confines QM/MM-specific logic to a dedicated module rather than distributing engine-specific calls throughout neighbor-searching, force accumulation, constraints, output, and free energy code paths.

**Figure 1:**
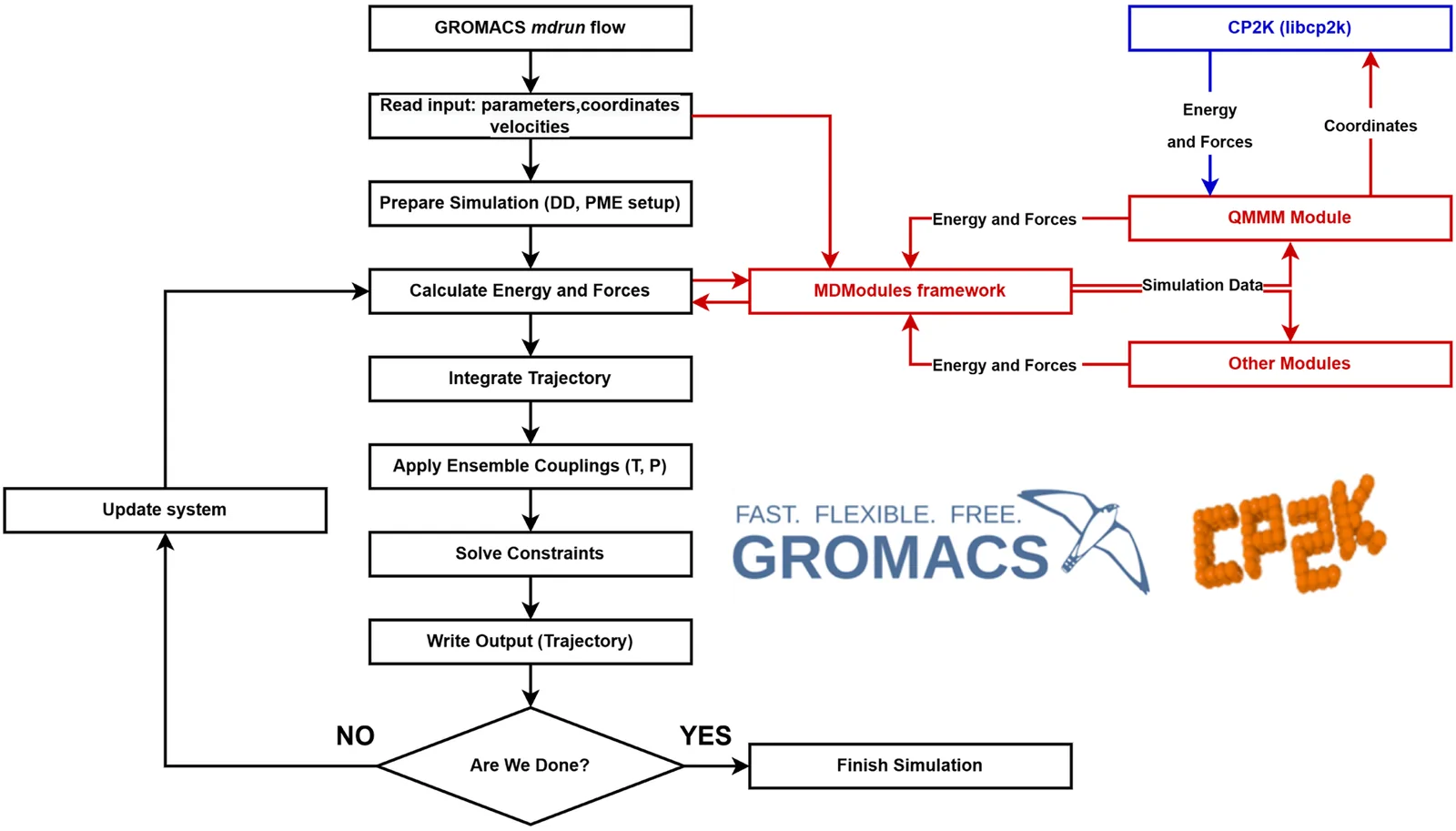
Software architecture of the GROMACS-CP2K interface. CP2K functionality (blue) is integrated into the GROMACS *mdrun* workflow (black) through the GROMACS MDModules layer (red). The module passes coordinates and cell information to CP2K, retrieves QM/MM energies and forces, and returns them to the ordinary GROMACS integration and sampling machinery.

The division of responsibilities was deliberate. CP2K owns the DFT problem, the GPW grids, GEEP electrostatic embedding, QM-cell decoupling, and QM/MM embedding forces. GROMACS owns the classical force field, constraints, time integration, trajectory handling, enhanced sampling, and an analysis ecosystem. At every step, the two codes exchange only the state variables needed for a Hamiltonian-consistent force evaluation: coordinates, cell information, QM/MM selections, energies, and forces. This division of responsibilities is the central design feature of the implementation: CP2K contributes the mathematically delicate fully periodic QM/MM electrostatics, while GROMACS contributes the mature simulation and sampling infrastructure that enables robust and reproducible QM/MM studies of complex biomolecular systems.

On the CP2K side, communication proceeds through *libcp2k*. A QM calculation is represented by a CP2K force environment created from a CP2K input file through cp2k_create_force_env. At every MD step, GROMACS updates this force environment by passing atomic positions and cell vectors with cp2k_set_positions and cp2k_set_cell, invokes the electronic structure energy and force evaluation through cp2k_calc_energy_force, and retrieves the resulting potential energy and atomic forces through cp2k_get_potential_energy and cp2k_get_forces. These calls keep the coupling at the level of stable public interfaces while leaving GPW grids, GEEP electrostatics, wave function optimization, and force assembly inside CP2K. As a result, the interface avoids file-based orchestration during dynamics and retains the tested numerical machinery of both codes.

The interface automates operations required when converting a fully classical GROMACS topology for use with the GROMACS-CP2K QM/MM coupling. The *grompp* preprocessor removes partial charges and Lennard-Jones (LJ) interactions within the QM region while retaining LJ interactions between QM and MM atoms. Bonded terms entirely inside the QM region are removed, and constraints involving QM atoms are disabled where necessary. Covalent QM/MM boundaries are capped in CP2K using link atoms. Each bond that crosses the QM/MM boundary is mapped to a CP2K *&LINK* section through the corresponding QM and MM atom indices. In the automatically generated input, CP2K applies its default IMOMM hydrogen cap, which the user could override in a custom input. The link coordinate is a dependent coordinate on the QM–MM bond, placed at *R*_link_ = *R*_QM_ + (*R*_MM_ – *R*_QM_)/α with the default α =1.38. It is reconstructed at every force evaluation and is not integrated as an independent GROMACS degree of freedom; its force is projected onto the real QM and MM boundary atoms. The link hydrogen is included in the CP2K Hamiltonian, including its basis and pseudopotential contributions and electronic density on the GPW grids, but has no corresponding MM point charge. The classical partial charge of the real MM boundary atom is retained by default. Boundary charge scaling or redistribution can be specified in a custom CP2K input. No additional compensating charge is introduced specifically for the link atom; the user-specified charge and multiplicity should refer to the complete hydrogen-capped QM subsystem. These operations are specific to the present interface and should not be understood as a standardized setup procedure across different QM/MM implementations.

The workflow can create a CP2K input file for common DFT settings or use a fully user-specified CP2K input file. This balance is intentional: routine cases should be reproducible without expert-level manual editing, while advanced users should retain complete control over the electronic structure setup.

### 2.4 Enhanced Sampling in QM/MM Simulations

Because CP2K is called from within the GROMACS force loop, QM/MM simulations can directly use enhanced sampling methods available in GROMACS. This is particularly valuable because many processes of interest in biomolecular QM/MM simulations, such as chemical reactions, conformational transitions, and proton-transfer events, occur on time scales that are inaccessible to straightforward molecular dynamics. Enhanced-sampling methods accelerate the exploration of configurational space and thereby enable the calculation of free-energy profiles and barriers at a substantially reduced computational cost.^8^

In this work, we employ both umbrella sampling (US)^22^ and accelerated weight histogram (AWH) methods.^13^ In US, a set of simulations restrains one or more collective variables at prescribed values, and the resulting free-energy profile is reconstructed using the weighted histogram analysis method (WHAM).^23^ In AWH, a bias potential is updated adaptively during the simulation to drive sampling toward a target distribution, from which the free-energy profile is obtained directly.^13^ Multiwalker AWH further accelerates convergence by sharing bias information among simulations initialized in different regions of the collective-variable space.

These adaptive sampling strategies are particularly attractive for QM/MM simulations, where each additional picosecond requires expensive electronic structure calculations, and reductions in the total trajectory length translate directly into improved computational efficiency. Further details of the AWH algorithm are provided in the original publication^13^ and the GROMACS documentation, while a detailed comparison between US and AWH can be found in Aho et al.^24^

## 3 Results

To assess the consistency of the energies and forces returned by the coupled implementation, we performed NVE simulations for *N*-Methylacetamide (NMA) in vacuum and for stilbene in water. The normalized total-energy drifts were −7.52 × 10^–5^ and 3.63 × 10^–4^ kJ mol^–1^ ps^–1^ atom^–1^ for the two NMA calculations and 7.01 × 10^–5^ kJ mol^–1^ ps^–1^ atom^–1^ for the hybrid stilbene system. Thus, after accounting for the different trajectory lengths and numbers of atoms, the drift in the solvated QM/MM system is of the same order as that observed in the all-QM vacuum tests. Representative NVE trajectories and the complete analysis are provided in Sections 3 and 4 of the Supporting Information.

After additional validation of our interface on reactive systems (see Supporting Information Sections 5 and 6), we demonstrate its application to a challenging QM/MM free-energy calculation: the phospho-transfer reaction catalyzed by the RNA-dependent RNA polymerase (RdRp) of SARS-CoV-2.^25^ This example was chosen because it combines several demanding aspects of biomolecular QM/MM simulations, including periodic boundary conditions, a large and heterogeneous molecular environment, multidimensional reaction coordinates, and an extensive enhanced sampling.

The incorporation of a nucleotide into the growing RNA strand involves a phosphotransfer reaction in which a nucleotide triphosphate undergoes nucleophilic attack by the terminal nucleotide of the RNA chain, accompanied by diphosphate release. Because bond formation and bond breaking occur concertedly, the reaction cannot be described adequately by a single geometrical coordinate. We therefore employed multidimensional AWH sampling in combination with the GROMACS-CP2K interface to compute the corresponding free-energy surface.

The starting structure was derived from the 6NUR crystal structure^26^ and completed using coordinates from the homologous polymerase complex, 3OL6,^27^ yielding a model of the RdRp active site with ATP positioned for incorporation into the growing RNA strand.

After the equilibration of the protein, we performed a two-dimensional free-energy surface simulation of the reaction along the d1 and d2 coordinates shown in Figure 2C. Details of these AWH simulations are provided in SI. The resulting free-energy surface is shown in Figure 3, both as a three-dimensional representation (A) and as a two-dimensional projection onto the reaction-coordinate plane (B). In both projections, the black arrow indicates the minimum free-energy pathway connecting the reactant and product states. The free-energy barrier along this reaction coordinate is approximately 90 kJ/mol with an estimated final target error of 1 kJ/mol, which is in good agreement with the barrier obtained from enhanced sampling along a one-dimensional collective variable, ξ defined as the difference between d2 and d1 (88.5 kJ/mol, SI). The free-energy difference between the reactant and product states is 39 kJ/mol.

**Figure 2:**
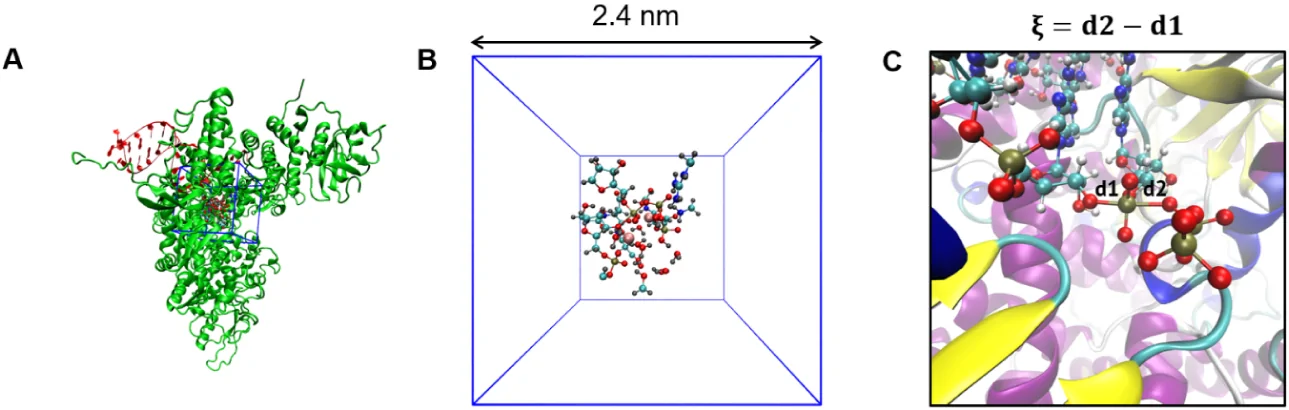
SARS-CoV-2 RNA-dependent-RNA-polymerase system used for reaction free-energy evaluation. Panel A shows the overall system: protein in green, RNA strands in red, and the QM cell in blue. A close look at the QM cell and QM atoms is shown in panel B. Reaction coordinated ξ definition is shown in panel C.

**Figure 3:**
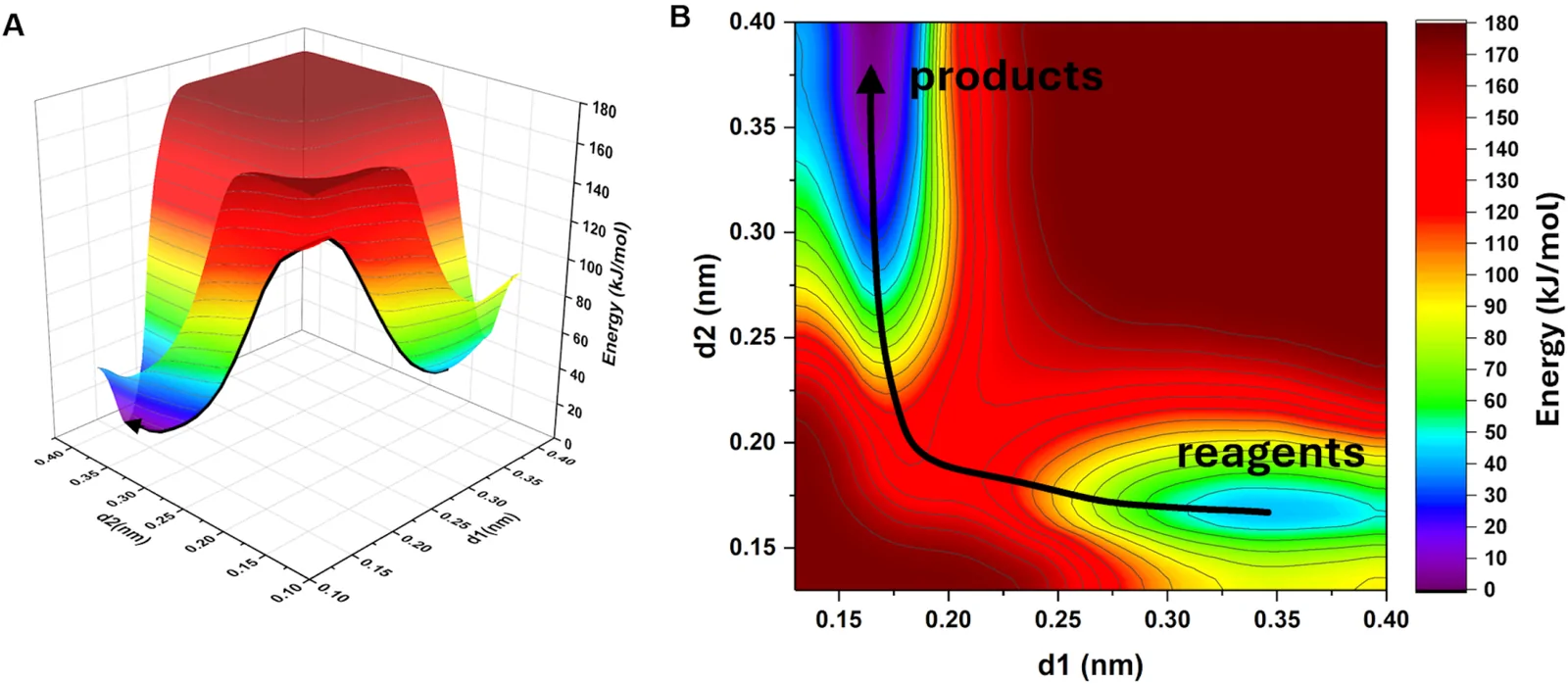
Free-energy surface of ATP incorporation into the RNA strand within the RdRp active site, obtained using the GROMACS-CP2K QM/MM interface combined with the two-dimensional AWH enhanced-sampling method. Panel A shows a three-dimensional representation, while panel B displays a two-dimensional color-coded map. The surface is projected onto the d1 and d2 reaction coordinates, defined in Figure 2C.

The RdRp calculation is intended to demonstrate the feasibility of the multidimensional multi-walker AWH sampling with the GROMACS-CP2K interface in a realistic enzymatic system. The resulting free energy surface displays an apparent barrier of approximately 90 kJ/mol along the selected phosphoryl transfer coordinates. These coordinates do not explicitly describe the transfer of the O3′ proton, and the surface is specific to the selected starting structure, protonation model, QM region, exchange–correlation functional, basis set, and sampling protocol. Because the sensitivity to these modeling choices was not investigated systematically, the calculated barrier should not be regarded as a definitive quantitative prediction of the RdRp catalytic barrier or mechanism. Consistent with this limitation, recent QM/MM studies using different reaction coordinates and proton transfer models reported substantially lower activation free energies.^28–30^ Although these values are not directly comparable because the calculations describe different chemical pathways and computational models, the discrepancy underscores the sensitivity of predicted enzymatic mechanisms and barriers to system preparation and reaction-coordinate definition.

## 4 Discussion

We have introduced a fully periodic, open-source QM/MM interface between GROMACS and CP2K. The implementation combines CP2K’s treatment of electrostatic embedding under periodic boundary conditions with the molecular-dynamics, enhanced-sampling, and analysis capabilities of GROMACS. The numerical tests support the consistent exchange of energies and forces between the two programs, while the application examples demonstrate that the interface can be used for QM/MM simulations ranging from small molecular systems to enzymatic reactions.

The energy conservation observed in the NVE tests provides a direct assessment of the consistency of the coupled energy and force evaluation. For electrostatic embedding, the interface combines CP2K’s GEEP treatment with decoupling and recoupling of the compact QM cell. Such a description avoids collocating every MM point-charge potential on the finest QM grid so that the dominant fine-grid embedding cost depends only weakly on the size of the surrounding MM environment, allowing the possibility to retain long-range periodic electrostatic interactions while limiting the additional cost associated with embedding large biomolecular environments.

Existing QM/MM software reflects different methodological and architectural priorities. General-purpose drivers such as QMMM 2023 provide access to several quantum-chemistry programs and a broad range of embedding, boundary treatment, and adaptive partitioning schemes.^31^ MiMiC instead employs a modular, loosely coupled intermediary framework in which client programs evaluate the different subsystem contributions.^32^ GROMACS or OpenMM can be used for the classical subsystem, and CP2K was recently added as a client for evaluating the quantum subsystem.^33^

MiMiC and the present interface also differ in their representation of the QM–MM electrostatic coupling. In MiMiC, MM atoms are divided into short- and long-range groups according to a user-defined cutoff. Nearby MM charges interact explicitly with the QM nuclei and electron density, whereas the interaction with more distant MM charges is evaluated using a multipole expansion of the QM charge distribution.^32,33^ The cutoff therefore does not truncate the electrostatic environment, but determines where the representation changes from explicit density-charge interactions to the long-range multipole approximation; its convergence is controlled by both the cutoff and the order of the multipole expansion. In the present interface, all MM charges instead contribute through a fully periodic potential representation, without dividing the environment into distance-based short- and long-range groups or replacing the interaction with distant MM charges. The two electrostatic schemes may differ in their numerical convergence and computational cost, but establishing an advantage in accuracy or time to solution would require controlled benchmarks employing equivalent Hamiltonians, boundary treatments, periodicity settings, and computational resources, which are beyond the scope of the present work.

In addition, the coupling architecture is tighter in the present interface: CP2K is called through *libcp2k* directly from the GROMACS force evaluation loop. GROMACS remains responsible for topology preprocessing, constraints, integration, trajectory output, sampling, and analysis, while CP2K evaluates the electronic structure, electrostatic embedding, and associated QM/MM forces. This architecture provides direct access to the native GROMACS workflow without requiring a separate coupling executable or intermediary framework but does not offer the engine interchangeability and independent resource allocation of MiMiC.

The tight integration also supports the usability and maintainability of the interface. Preprocessing operations required to convert a classical GROMACS topology for use with the GROMACS-CP2K coupling are automated, while advanced users can retain full control over the electronic-structure setup by supplying a customized CP2K input. Because the interface is maintained within the GROMACS source tree and accesses CP2K through its standard library interface, compatibility can be addressed through the established development and release processes of the two packages. Documentation,^34^ tutorials,^35^ training schools,^36^ and recorded lectures^37,38^ are available to support reproducible use of the software.

The application examples show that native GROMACS enhanced sampling methods, including umbrella sampling and multidimensional, multiwalker AWH, can be applied to QM/MM systems through the interface. Their quantitative results nevertheless remain dependent on the selected reaction coordinates, the QM region, the electronic structure method, the system preparation, and the sampling protocol. They should therefore be interpreted primarily as demonstrations of the capabilities of the implementation rather than as definitive reference calculations for the respective chemical reactions. The specific limitations of the RdRp free-energy surface are discussed in the Results section.

The most immediate route for extending the range of applications is through additional electronic structure methods implemented in CP2K and sampling methods available in GROMACS. Recent CP2K developments enable GEEP-style MM-charge embedding with SCC-DFTB, GFN1-xTB, and GFN2-xTB, providing lower-cost alternatives to density-functional QM/MM calculations. These developments complement recent GFN1-xTB benchmarks for liquid water^39^ and the implementation of periodic GFN2-xTB in CP2K.^40^ The GROMACS-CP2K interface therefore provides a practical and community-maintained foundation for reproducible multiscale simulations of chemical reactivity in complex systems.

## Supplementary Material



## Data Availability

All simulations models, input files, and structures used in the work are available for download from the Zenodo research data storage.^41^ The interface is available in release versions of GROMACS (www.gromacs.org, version 2022 or later) and CP2K (www.cp2k.org, version 8.1 or later).

## Notes

Basic installation instructions, reproducibility checklist, and all details of all performed simulations: validation for NMA in vacuum under NVE conditions; stilbene in water under NVE, NVT, and NPT conditions; Diels–Alder reaction energetics simulations with relaxed-surface, umbrella sampling, and AWH; a comparison to previous CP2K QM/MM umbrella-sampling simulations of phytochrome chromophore isomerization in protein; and details of free-energy surface simulation with umbrella sampling and AWH for ATP incorporation by SARS-CoV-2 RdRp (PDF)
