# Supplementary material for: Seamless QM/MM Simulations via a GROMACS-CP2K Interface
Source: J Chem Inf Model. 2026 Sep 10;66(18):11588–95. doi: 10.1021/acs.jcim.6c02073 (PMC13625966; doi:10.1021/acs.jcim.6c02073)
Supplement: Supplementary file 1 [file ci6c02073_si_001.pdf]

Supporting information for Seamless QM/MM simulations via a GROMACS–CP2K interface

Dmitry Morozov,[*,†] Christian Blau,[‡] Ole Schütt,[¶] Vladimir Mironov,[§] Arno Proeme,[‖] Holly Judge,[‖] Thomas D. Kühne,[⊥,#,@] Berk Hess,[‡] and Gerrit Groenhof[*,†]

†*Nanoscience Center and Department of Chemistry, University of Jyväskylä, P.O. Box 35, FI-40014 Jyväskylä, Finland*

‡*Department of Applied Physics, Science for Life Laboratory, KTH Royal Institute of Technology, SE-171 65 Solna, Sweden*

¶*CP2K Foundation, CH-8057 Zürich, Switzerland*

§*Terra Quantum AG, Kornhausstrasse 25, CH-9000 St. Gallen, Switzerland*

‖*EPCC, The University of Edinburgh, Bayes Centre, 47 Potterrow, Edinburgh EH8 9BT, United Kingdom*

⊥*Center for Advanced Systems Understanding (CASUS), Untermarkt 20, 02826 Görlitz, Germany*

#*Helmholtz-Zentrum Dresden-Rossendorf (HZDR), Bautzner Landstraße 400, 01328 Dresden, Germany*

@*Institute of Artificial Intelligence, Technische Universität Dresden, Helmholtzstraße 10, 01069 Dresden, Germany*

E-mail: dmitry.morozov@jyu.fi; gerrit.x.groenhof@jyu.fi

Contents

1 Installing GROMACS-CP2K **S3**

2 Reproducibility Checklist **S3**

3 N-Methylacetamide (NMA) **S4**

3.1 Simulation details S4

3.2 Results S5

4 Stilbene molecule in water **S6**

4.1 Simulation details S6

4.2 Results S7

5 Diels-Alder reaction **S9**

5.1 Simulation details S9

5.2 Results S10

6 Phytochrome isomerization **S11**

6.1 Simulation details S11

6.2 Results S12

7 RNA-dependent RNA polymerase (RdRp) free energy of ATP Incorporation **S14**

7.1 Simulation details S14

7.2 Results S16

References **S17**

1 Installing GROMACS-CP2K

The interface is distributed as part of the GROMACS code base and can be installed either through the Spack package manager[1] or by compiling GROMACS against the *libcp2k* library provided by CP2K.[2,3] Detailed installation instructions and example build configurations are provided in the online documentation.

The recommended way of installing GROMACS with CP2K is through the Spack package manager[1]. After installing Spack itself[4], the two combined software packages can be installed with a single command: `spack install gromacs+cp2k`.

An installation without Spack starts with building CP2K[2]. Since *libcp2k* is included with CP2K by default, no special settings are needed. Thereafter, GROMACS can be built[3]. When invoking CMake, support for CP2K is enabled via the flag `-DGMX_CP2K=ON`, and the installation path of *libcp2k* has to be passed via `-DCP2K_DIR=<path>`. Afterward, the installation of GROMACS can proceed as usual. This will result in a `gmx` binary that is linked against the *libcp2k* library to enable the GROMACS-CP2K interface.

2 Reproducibility Checklist

For each QM/MM calculation, a complete reusable setup consists of the GROMACS molecular topology and coordinate files, the molecular-dynamics parameter file, the QM atom selection, any link-atom definitions at covalent QM/MM boundaries, the CP2K input file, and the collective-variable or pull-coordinate definitions used for umbrella sampling or AWH. The preprocessing step removes intra-QM force-field terms and charges from the GROMACS topology, retains QM–MM Lennard–Jones interactions, and writes the CP2K input used to initialize the *libcp2k* force environment. For production calculations, we recommend archiving both the generated CP2K input and the processed GROMACS topology, because these files define the Hamiltonian actually used in the coupled simulation.

The main-text examples use GEEP electrostatic embedding, compact CP2K QM cells embedded

in larger periodic GROMACS cells, and explicitly reported reaction-coordinate definitions. Repeating a calculation therefore requires preserving the QM cell dimensions, MM periodic cell, GEEP settings, basis set, density functional, thermostat or barostat settings, sampling window centers or AWH ranges, bias force constants, and discarded equilibration intervals. Processed data underlying all free-energy profiles should be distributed together with the raw input files so that readers can regenerate the figures without rerunning the full QM/MM trajectories.

For clarity, the GEEP orders quoted below refer to the value of the CP2K input keyword `USE_GEEP_LIB`, i.e., the number of terms selected from the internal GEEP library. This order is common to all MM atom kinds in a calculation, while the atom-kind-dependent smoothing radii rescale the Gaussian coefficients and widths. The quoted input order should not be interpreted as a count of any additional historical compatibility-correction term that CP2K may generate internally, depending on the input settings.

3 N-Methylacetamide (NMA)

3.1 Simulation details

N-Methylacetamide (NMA) is a small molecule characterized by a single peptide bond and two methyl groups. It is frequently utilized as a test case in computational studies[5,6]. In this work, we employ NMA in conjunction with two different box sizes to ensure total energy conservation within our QM/MM interface during NVE molecular dynamics (MD) simulations. The first box size measures 1x1x1 nm, which is consistent for both CP2K and GROMACS, as illustrated in Fig. S1A. The second box size is significantly larger, measuring 10x10x10 nm, and encompasses the entire QM/MM system. However, in the CP2K quantum mechanical cell, where the wavefunction is expanded on a multigrid, in the simulation kept at a size of 1x1x1 nm, as depicted in Fig. S1B. For both cases we employed PBE[7] functional and DZVP-MOLOPT-GTH[8] basis set.

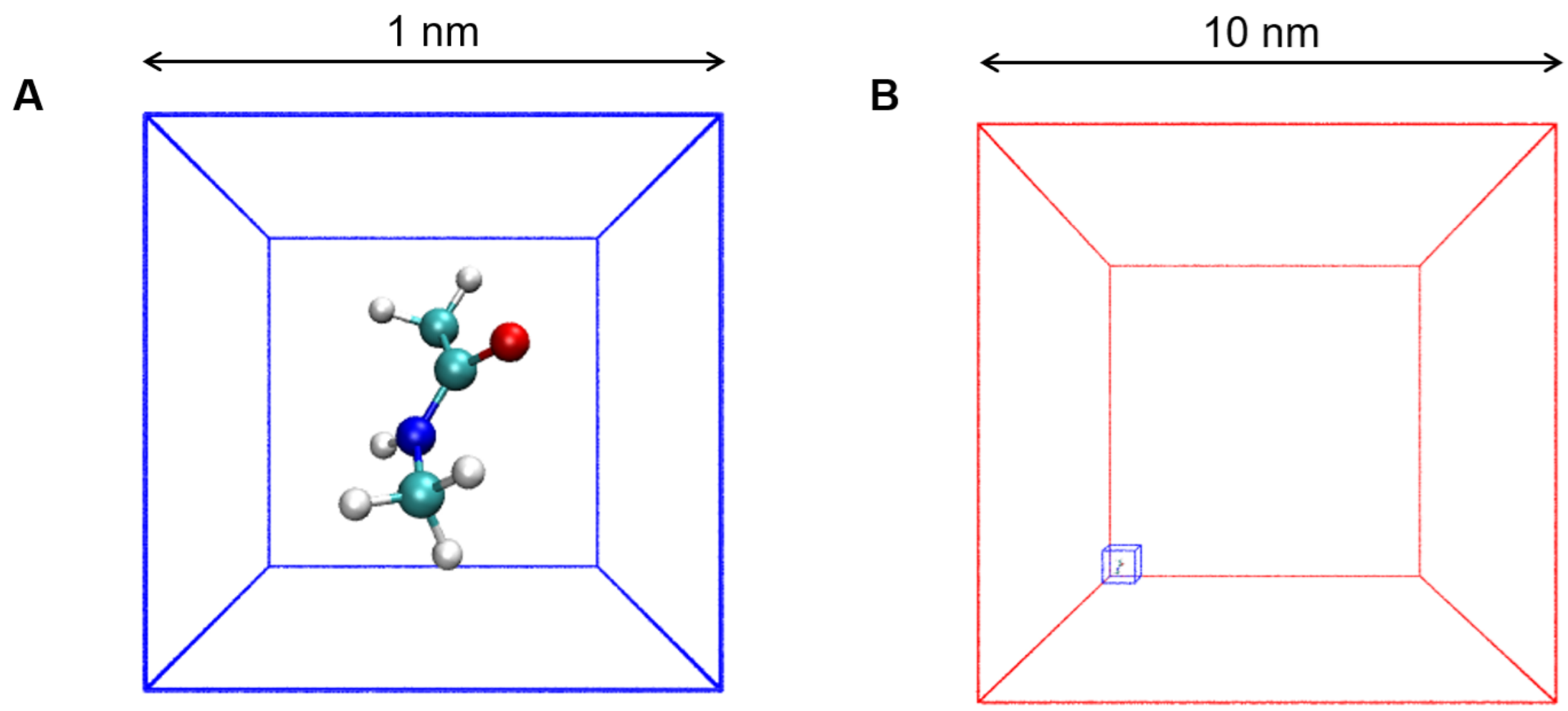

Figure S1: The NMA molecule system utilized for NVE simulations with two box sizes. The small 1x1x1 nm box is shown in panel A, while the large 10x10x10 nm QM/MM box with the small 1x1x1 nm cell is presented in panel B.

3.2 Results

The NVE simulation results for the NMA molecule in a vacuum, utilizing our GROMACS-CP2K QM/MM interface, are illustrated in Fig. S2. In both test simulations, the system exhibits minimal drift in total energy relative to the initial structure at the 0 ps timepoint. Linear fits to the total energy, normalized by the trajectory length and number of atoms, give drift rates of approximately -7.52×10^{-5} kJ mol^{-1}ps^{-1}atom^{-1} for the small box and $+3.63 \times 10^{-4}$ kJ mol^{-1}ps^{-1}atom^{-1} for the large box. The RMSD of energy fluctuations remains below 0.5 kJ/mol over a 50 ps trajectory, indicating overall high stability throughout the simulation. Additionally, to achieve such exceptional energy conservation performance, it is necessary to disable the center of mass motion removal feature in GROMACS by setting the "*comm-mode* = *None*" mdp option. This requirement can be attributed to the grid nature of the CP2K wavefunction (and consequently energy), which is not invariant to external translations of the system.

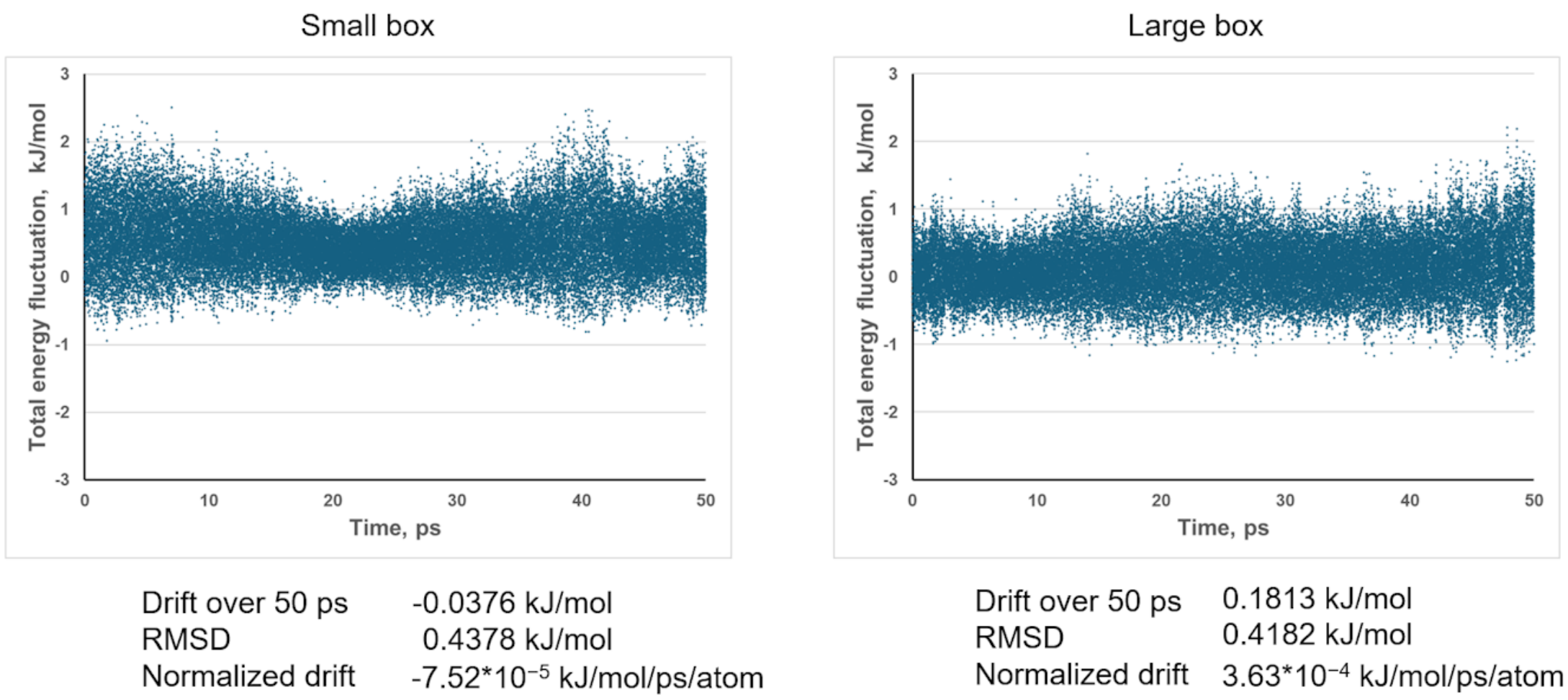

Figure S2: Total energy fluctuations with respect to the initial structure of two NMA NVE simulations. Total energy drift and RMSD over the 50 ps trajectories are show below.

4 Stilbene molecule in water

4.1 Simulation details

The NMA system, presented above, utilized only QM atoms, which meant that no QM/MM interactions were involved in the simulations. To enhance this approach, we evaluated a different model molecule, trans-stilbene, at the QM level within a box of water molecules, modeled using TIP3P forcefield. Similar to the previous system, we employed two boxes: a smaller one referred to as the QM cell, shown in blue on Fig. S3, and a larger QM/MM box that encompasses the entire system, shown in red on Fig. S3. Our objective for this system was not only to demonstrate energy conservation in our QM/MM interface implementation but also to showcase its ability to manage constant temperature (NVT) and pressure (NPT) ensembles, utilizing standard coupling schemes available in GROMACS. In all simulations we have used a leap-frog integrator with 1 fs time-step and SETTLE constraints for water molecules. Temperature for both NVT and NPT simulations have been set to 300K using v-rescale thermostat with 0.1 ps time constant. The c-rescale barostat with 0.1 ps time constant has been employed to keep the pressure of 1 bar in the NPT simulation. For all cases QM subsystem has been described at the PBE/DZVP-MOLOPT-GTH[7,8] level of theory and GEEP electrostatics with 12 gaussians per charge[9] have been applied to account for the interactions

between QM and MM subsystems.

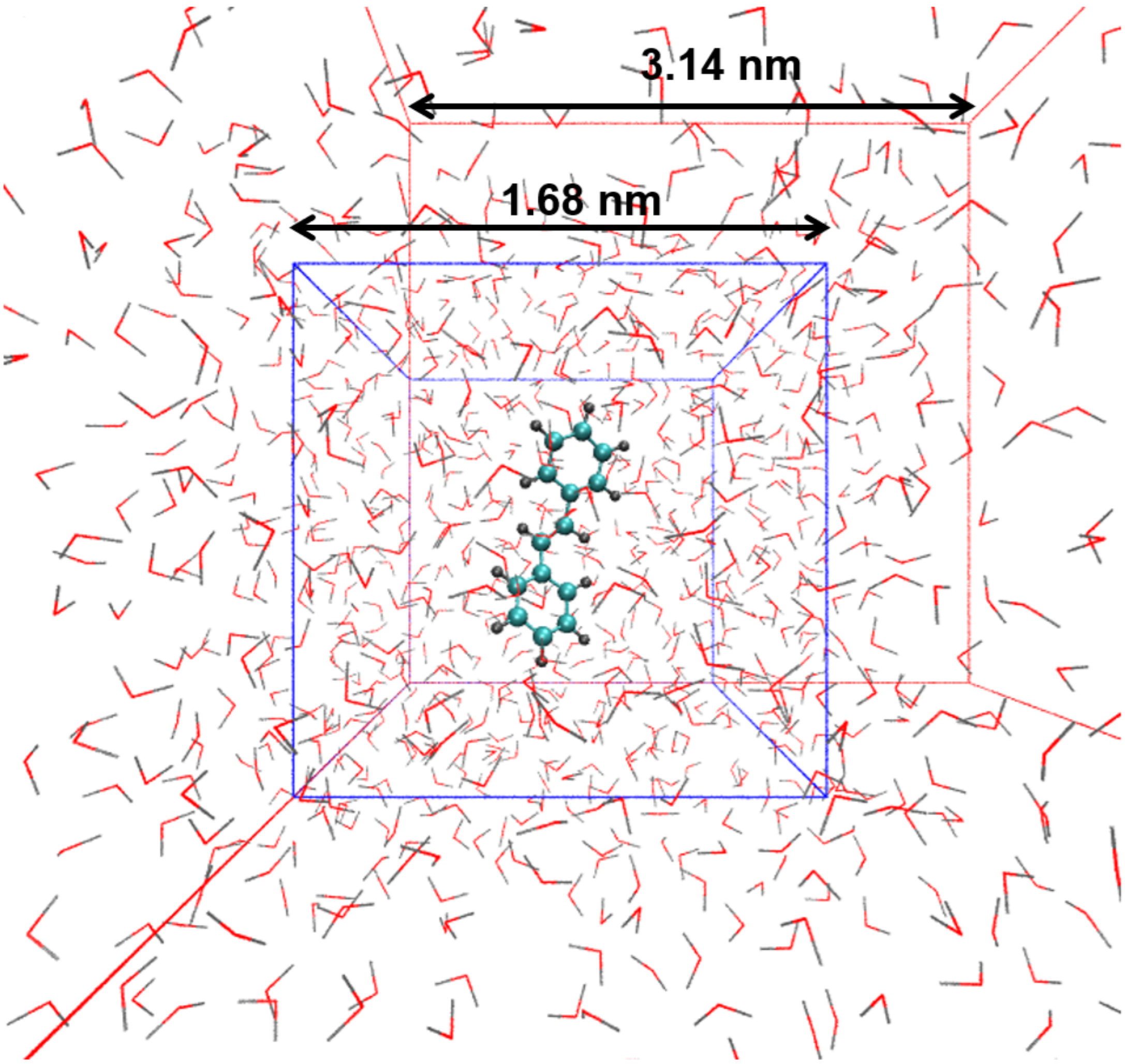

Figure S3: Stilbene in water system, with the sizes of the large QM/MM box (red) and small QM cell (blue) indicated in nanometers.

4.2 Results

The simulation results for the stilbene (QM) molecule in a TIP3P, utilizing our GROMACS-CP2K QM/MM interface, are illustrated in Fig. S4. In the NVE simulation we observe overall drift higher than in previous NMA simulations. Normalizing the linear energy-drift estimate by both trajectory length and number of atoms gives approximately $+7.01 \times 10^{-5}$ kJ mol^{-1}ps^{-1}atom^{-1}. This is of the same order as the small-box NMA result and below the corresponding large-box NMA value.

Because the systems and trajectory lengths differ, this comparison does not by itself identify a system-specific source of the drift. In both NVT and NPT simulations temperature coupling shows very nice average temperature around 300K with the maximum magnitude of fluctuations around 15K. For the pressure coupling simulation shows high fluctuations of the total pressure, however on average pressure is very close to the target value of 1 bar, especially considering that for now we do not account for the force virial due to QM-MM interactions.

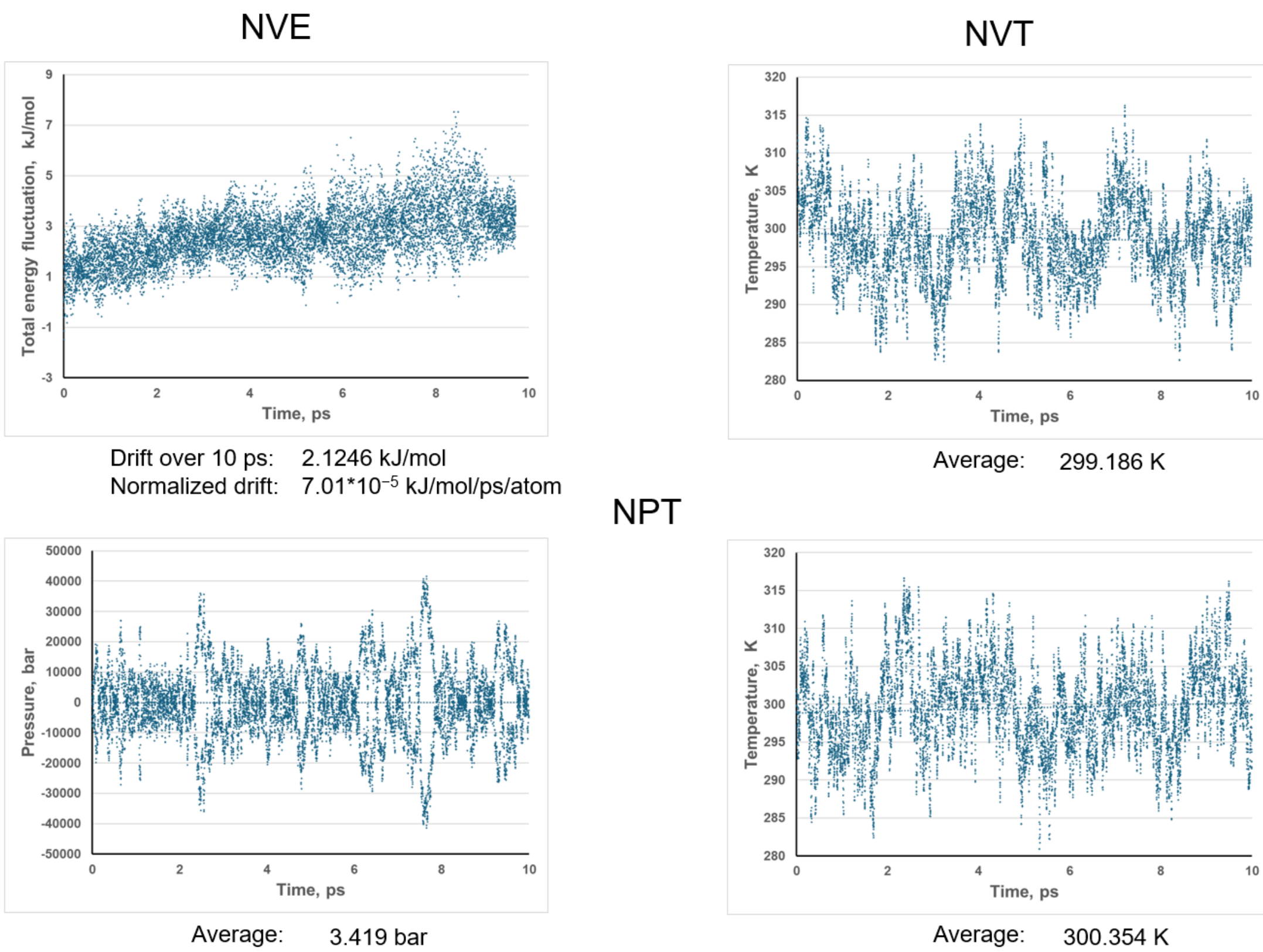

Figure S4: Simulation results for stilbene in water system. NVE simulation is on the top-left panel, NVT simulation is on the top-right panel and NPT simulation (pressure and temperature) is on the bottom panels.

5 Diels-Alder reaction

5.1 Simulation details

After we complete the basic tests, we progress towards the study of chemical reactivity. The first system we selected is the reaction that takes place inside the catalytic antibody 1E9, which can deactivate harmful substances by catalyzing the Diels-Alder reaction. We utilized the crystal structure 1C1E from the PDB database, which presents the products of this reaction (Fig. S5A), as revealed by Xu et. al.[10] We extracted the product from the crystal, divided it into QM and MM parts, as shown on the Fig. S5B. The total QM/MM box was maintained at approximately five times the size of the QM cell, shown on Fig. S5C. The purpose of this test case is to demonstrate GROMACS capability, in conjunction with our QM/MM interface, to simulate the energy profiles of such reactions using various techniques. In this study, we compared the relaxed potential energy surface with the free-energy surfaces obtained from umbrella sampling and accelerated weight histogram (AWH) simulations[11,12]. For the free-energy simulations we have used NVT ensemble at 300K with v-rescale thermostat[13] and leap-frog integrator with 1 fs time-step. For QM we used DFT theory at the PBE/DZVP-MOLOPT-GTH[7,8] level and GEEP electrostatics with 12 gaussians per charge[9] have been applied to account for the interactions between QM and MM subsystems. The reaction coordinate is defined as the distance between the centers of two reacting bonds, as illustrated in Fig. S5C with a red arrow. To construct the relaxed potential energy surface (PES), we conducted constrained geometry optimization using the steepest descent method at 67 points along the reaction coordinate, ranging from 0.12 nm to 0.45 nm with a step size of 0.05 nm. To constrain the reaction coordinate, we introduced two bonds between the respective carbon atoms that form during the reaction and fixed them for each relaxed surface point using the LINCS algorithm[14].

For the umbrella sampling simulation, we established 21 points along the reaction coordinate from 0.15 to 0.53 nm, spaced 0.02 nm apart. We performed a 10 ps NVT simulation at 300 K for each point, restraining the coordinate with a harmonic potential that had a force constant of 128000 kJ $mol^{-1}nm^{-2}$. The free-energy profile was subsequently convolved using the GROMACS WHAM

utility[15].

In the AWH simulation, we conducted 50 ps of sampling along the reaction coordinate range of 0.13 to 0.45 nm, applying a force constant of 128000 kJ $mol^{-1}nm^{-2}$ for the biasing harmonic potential and a coordinate diffusion constant of 0.001 nm^2ps^{-1}.

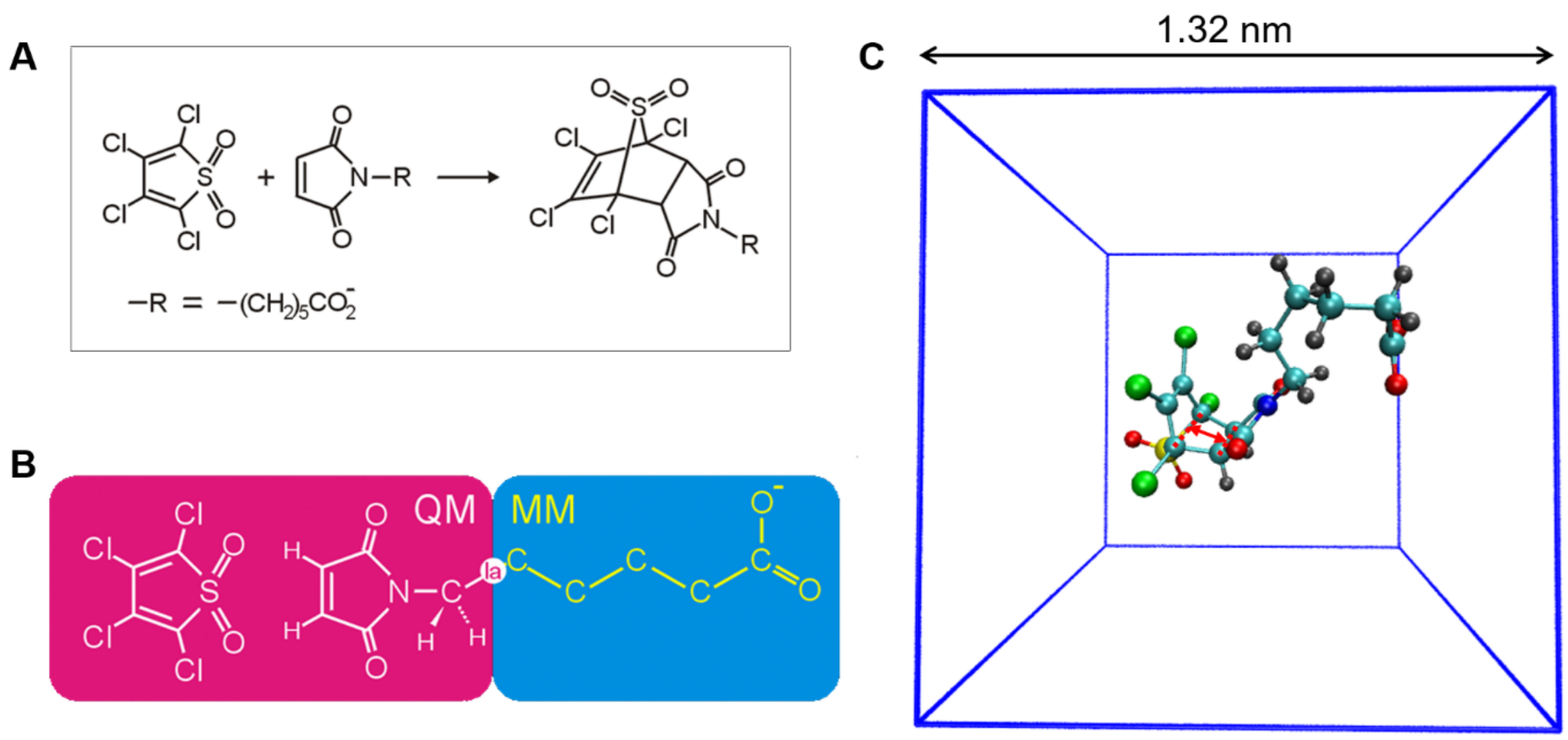

Figure S5: Diels-Alder reaction (panel A) products extracted from the crystal structure 1C1E. Split between QM and MM regions is shown on panel B. QM cell setup is shown on panel C.

5.2 Results

The energy surfaces derived from all three methods are illustrated on Fig. S6. Notably, all methods consistently reveal a minimum energy geometry (products of the reaction) with a reaction coordinate of 0.149 nm and a transition state at 0.23 nm. In contrast, the relaxed Potential Energy Surface (PES) estimates a barrier of 61.5 kJ/mol, with the reagents region approximately 135 kJ/mol higher than the product states, whereas both the US and AWH methods yield a consistent barrier of 87 kJ/mol with the reagents region of 100 kJ/mol, highlighting the significance of the entropic factor and the differences between potential energy and free-energy surfaces. Bootstrap analysis for up-to 100 attempts of the US data gave a maximum point-wise standard error of 3.81 kJ/mol and an activation barrier standard error of 2.6 kJ/mol, whereas the internal AWH target error estimate decreased to 3.83 kJ/mol after 50 ps. Because these quantities are obtained using different estimators, they

should not be interpreted as equivalent standard errors. Nevertheless, the stability of the profiles with increasing sampling supports convergence at an approximation resolution of 4 kJ/mol (∼1 kcal/mol). AHW required 50 ps of aggregated QM/MM sampling, compared to 10 ps * 21 windows = 210 ps for US. The more detailed comparison of AWH and US methods were performed in the work by Aho et.al.[12]

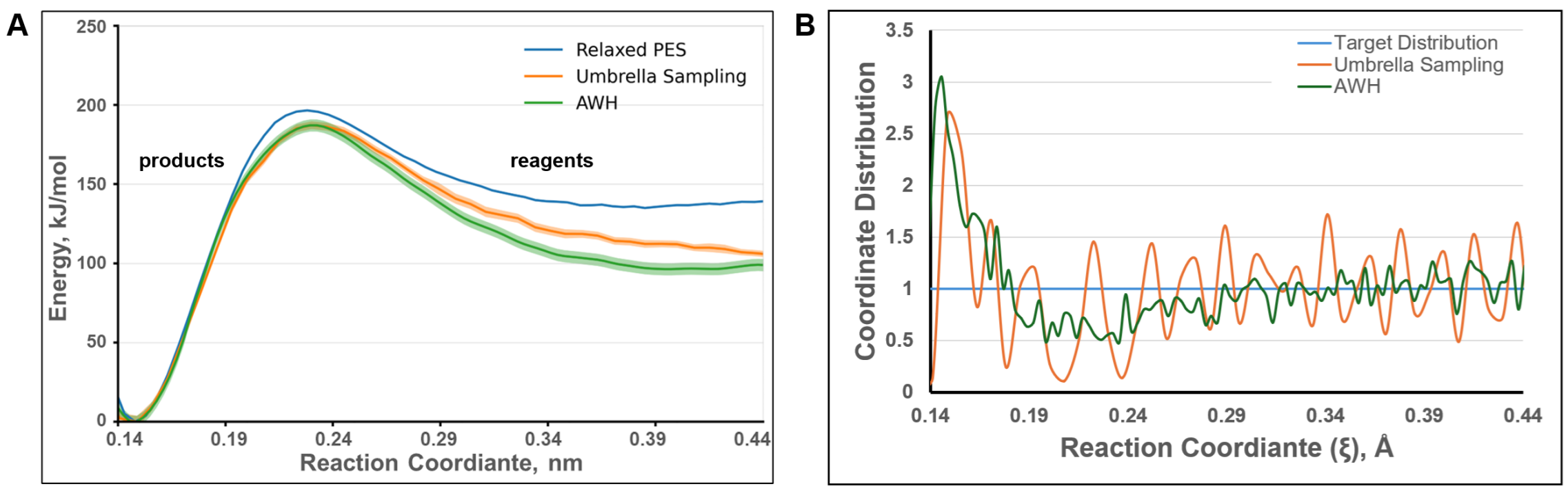

Figure S6: Panel A presents Diels-Alder reaction energy profiles obtained by relaxed PES scan, US and AWH simulations. Standard error of US, evaluated with bootstrapping, and estimated target error of AWH is shown as a margins on the corresponding graph. Panel B shows sampled with US and AWH reaction-coordinate distributions.

6 Phytochrome isomerization

6.1 Simulation details

To demonstrate the capabilities of our GROMACS-CP2K interface in simulating reactions within complex biological systems, we decided to replicate simulations from our previous work on the *Deinococcus Radiodurans* phytochrome[16]. In that study, we utilized CP2K's QM/MM module to simulate the thermal isomerization of the chromophore from Lumi-R to Meta-R states throughout the photocycle. Our objective in the current work is to evaluate the free energy profile associated with this isomerization using the umbrella sampling methodology and to compare the results of our GROMACS-CP2K QM/MM interface with those from earlier simulations (see section "QM/MM umbrella sampling simulations" of the Supporting Information and Figure S8(c) from previous work[16]. For that purpose we utilsed the QM/MM model of the system shown on Fig. S7A with QM

cell and subsystem of atoms and reaction coordinate shown on Fig. S7B. While, the full details of the system preparation and intial simulations setup could be found in our previous paper[16], umbrella sampling simulations setup have been simplified. Starting from the initial strucutres for all 13 umbrella windows we have performed 35-40 ps of QM/MM dynamics at PBE-D3/DZVP-MOLOPT-GTH//Amber03[7,8,17,18] level of theory and GEEP electrostatics with 8 gaussians per charge[9] emplyoing velocity-Verlet integrator with 1 fs timestep and NVT ensemble at 300K using v-rescale thermostat with 0.1ps time constant. First 5 picoseconds of within each window were considered as an equilibration time and thus excluded from the further analysis. The free-energy profile and histogram was convolved using the GROMACS WHAM utility.

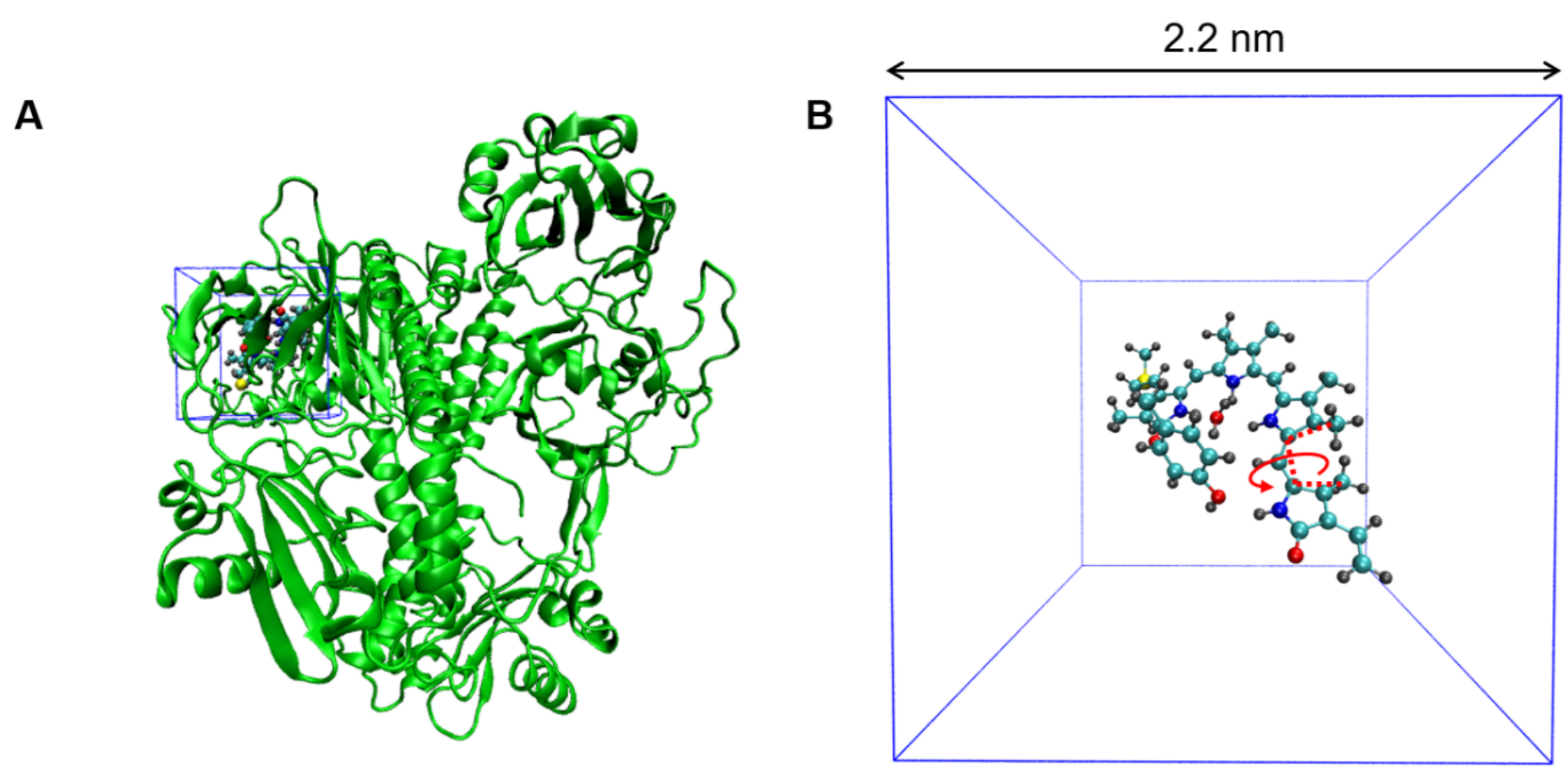

Figure S7: *Deinococcus radiodurans* phytochrome system used for the simulations of isomerization. Full QM/MM system is shown on panel A, with close look into the QM cell and QM atoms subsystem shown on panel B. Red dotted lines and arrow are showing dihedral angle employed as a reaction coordinate.

6.2 Results

The simulation utilizing our new QM/MM interface demonstrates a high degree of consistency with our previous results[16]. The comparison of the obtained free-energy profiles is illustrated in Fig. S8A. Our new interface indicates a slightly elevated reaction barrier of 36 kJ/mol, compared to 33 kJ/mol in the previous CP2K simulation. However, the free-energy difference between

reactants and products remains nearly identical at 26 kJ/mol. The distributions of the reaction coordinate within each window are depicted in Fig. S8B, revealing that the individual umbrellas exhibit sufficient overlap, thus ensuring the reliability of the free-energy profile. The standard errors of activation free-energy barriers, calculated with 100 bootstrapping attempts, for both simulations is very similar as well. We estimate it at 0.8 kJ/mol for CP2K and 1.0 kJ/mol for GROMACS-CP2K, while the the maximum point-wise standard error of the profiles estimated at 1.3 kJ/mol for CP2K and 1.66 kJ/mol for GROMACS-CP2K simulations respectively. Overall, we confirmed that our new GROMACS-CP2K QM/MM interface could be used to study the reactions within biological systems employing standard umbrella sampling protocol in combination with tools, such as WHAM, already available within the GROMACS package.

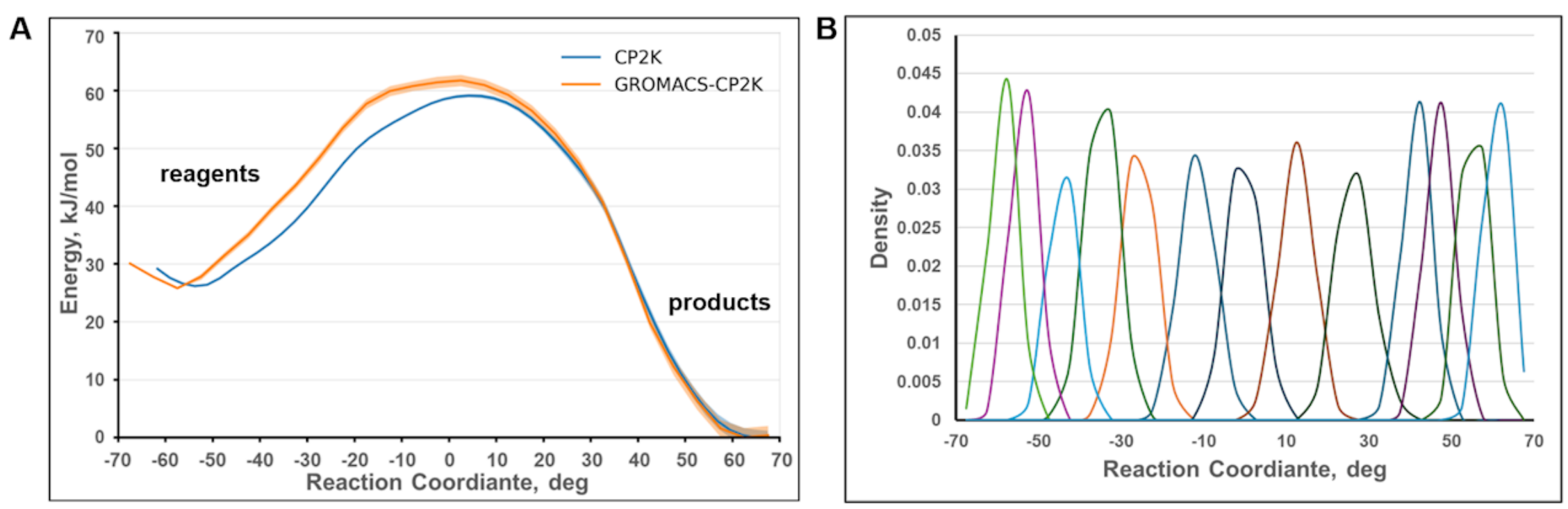

Figure S8: Panel A presents a comparison of the free-energy profiles obtained in the current study using the GROMACS-CP2K QM/MM interface (orange) against those from the previous study with CP2K (blue). Standard error of US, evaluated with bootstrapping, is shown as a margins on the corresponding graph Panel B displays the density of the reaction coordinate distribution within each window, as obtained with the GROMACS-CP2K QM/MM interface.

7 RNA-dependent RNA polymerase (RdRp) free energy of ATP Incorporation

7.1 Simulation details

The final system to which we will apply our QM/MM methodology is the RNA-dependent RNA polymerase (RdRp) of the SARS-CoV-2 virus. The incorporation of the next nucleotide into the viral RNA strand involves a complex phosphotransfer reaction, where a nucleotide triphosphate (NTP) undergoes a nucleophilic reaction with the preceding nucleotide in the growing RNA strand, resulting in the release of diphosphate. Typically, the reaction coordinate in such systems cannot be described by a single distance but rather as a combination of several distances. In this study, we utilize our QM/MM interface alongside GROMACS capabilities to manage complex reaction coordinates and enhanced sampling techniques (US and AWH) to simulate the free-energy profile associated with the incorporation of ATP nucleotide into the RNA strand.

The starting structure for our simulations was constructed from the 6NUR crystal structure[19] with a template RNA strand of 18 nucleotides (5'-CCAGUUCUCUCGUCCGGA-3') and growing complimentary RNA strand of 14 nucleotides (5'-GCCCGGACGAGAGA-3') from the homological polymerase complex 3OL6[20] and inserted into the RdRp protein. The adenosine triphosphate (ATP) reactant molecule was positioned in the active site adjacent to the uracil nucleotide from the complementary RNA strand. Missing protein loops and side chains were reintroduced using Modeller 9.22[21]. The Amber14sb force field[22] was employed for the protein, RNA, ATP, and ions. The entire system was immersed in a 15x15x15 nm cube of TIP3P water[23], with sodium and chloride ions added to neutralize the system and achieve a total salt concentration of 0.1M. Subsequently, the system was minimized using the steepest descent optimizer and equilibrated for 100 ns using the NVT ensemble at 300K with a v-rescale thermostat[13].

The final classical MD frame was converted to a QM/MM model (see Fig. 2 of the main text). The QM/MM system was equilibrated for 10 ps using the PBE functional with D3 dispersion

corrections,[7,17] a DZVP-MOLOPT-GTH[8] basis set and GEEP electrostatics with 8 gaussians per charge[9] employing velocity-Verlet integrator with 1 fs timestep and NVT ensemble at 300K using v-rescale thermostat with 0.1 ps time constant. Following equilibration, a pulling simulation was executed along the reaction coordinate $\xi = d2 - d1$ depicted in Fig. 2C. The reaction coordinate was pulled from -0.16 nm to 0.2 nm (from reagents to products) over the 10 ps QM/MM MD trajectory, which corresponds to 0.036 nm/ps rate. GROMACS transformation coordinate capabilities for pulling were used to define the reaction coordinate. A pulling simulation generated starting structures along a transformation coordinate describing ATP incorporation. From the pulling trajectory, 16 starting structures for umbrella sampling windows were extracted with reaction coordinate values of: -0.16, -0.13, -0.10, -0.07, -0.05, -0.03, -0.01, 0.00, 0.01, 0.03, 0.05, 0.07, 0.10, 0.13, 0.16, and 0.19 nm. Umbrella sampling QM/MM MD simulations were conducted in each window for 40 ps with 1 fs timestep and NVT ensemble at 300K using v-rescale thermostat with 1 ps time constant. A force constant of 128000 $\text{kJmol}^{-1}\text{nm}^{-2}$ was employed for the harmonic biasing potential. The total free-energy profile was convolved using the WHAM tool from the GROMACS package[15]. The first 5 picoseconds of simulation within each window were considered as an equilibration time and thus excluded from the further analysis. For comparison, we also performed AWH simulations of the free-energy profile using six walkers[11]. Each walker was initiated from structures obtained from the pulling simulation at -0.16, -0.10, -0.5, 0.07, 0.13 and 0.19 nm. Each walker ran for 25 ps, with all walkers coupled through a multi-walker scheme that exchanged and synchronized their biases every 100 fs. A force constant of 96000 $\text{kJmol}^{-1}\text{nm}^{-2}$ for the biasing harmonic potential and a coordinate diffusion constant of 0.001 $\text{nm}^2\text{ps}^{-1}$ were used for AWH sampling. The total convolved free-energy potential was extracted using the AWH tool from the GROMACS package.

In addition, we performed a two-dimensional free-energy surface simulation of the reaction along the d1 and d2 coordinates shown in Fig. 2C of the main text. In total, six walkers were used: three initialized in the vicinity of the reactant state and three near the product state. The starting points were identical to those used in the 1D AWH simulation, namely -0.16, -0.10, -0.5, 0.07, 0.13 and 0.19 nm along the collective reaction coordinate. Each walker was propagated for

50 ps, and all walkers were coupled through a multi-walker scheme, exchanging and synchronizing their bias potentials every 100 fs. A force constant of 96000 kJmol^{-1}nm^{-2} was employed for the harmonic biasing potential, together with a coordinate diffusion constant of 0.001 nm^{2}ps^{-1} for AWH sampling. Sampling of both d1 and d2 coordinates was performed over the range of 0.13 to 0.40 nm. The two-dimensional free energy surface was interpolated using a bicubic spline. After identifying the saddle point separating the reactant and product basins two downhill branches were initiated on opposite sides of the saddle point and traced by integrating the normalized negative gradient of the interpolated surface until the corresponding free energy minima were reached. The two branches were then joined at the saddle point to obtain the approximate minimum free energy path shown on Fig. 3 of the main text.

7.2 Results

Both AWH and US methods exhibit very similar free-energy profiles (see Fig. S9A), with reaction barriers of 93.5 kJ/mol for umbrella sampling and 88.5 kJ/mol for AWH. The products show slightly different energy levels: -42.2 kJ/mol for umbrella sampling and -30.9 kJ/mol for AWH. For US simulation standard error, calculated with 100 bootstrapping attempts, for barrier is estimated at 3.8 kJ/mol with the maximum point-wise profile standard error is 6 kJ/mol. For AWH simulation reported target error, used as an internal convergence criteria, at the end of simulation is estimated at 2.9 kJ/mol for 1D AWH and 1kJ/mol for 2D AWH. The major differences can be also observed in the coordinate sampling distribution plot in Fig. S9B. Umbrella sampling (orange line) oversamples regions near the middle of the umbrella windows but undersamples the most important regions along the intersections of the umbrella windows. In contrast, AWH aims to push the overall coordinate distribution towards the target distribution, which in this case was defined as a straight unitary line. Consequently, AWH typically requires much less overall sampling to gather the necessary statistics and converge the free-energy profile. In this particular case, for umbrella sampling, we conducted 16 windows $\times$ 40 ps = 640 ps of QMMD simulations, while AWH utilized only 6 walkers $\times$ 25 ps = 150 ps of QMMD simulations in total, achieving quantitatively the same result.

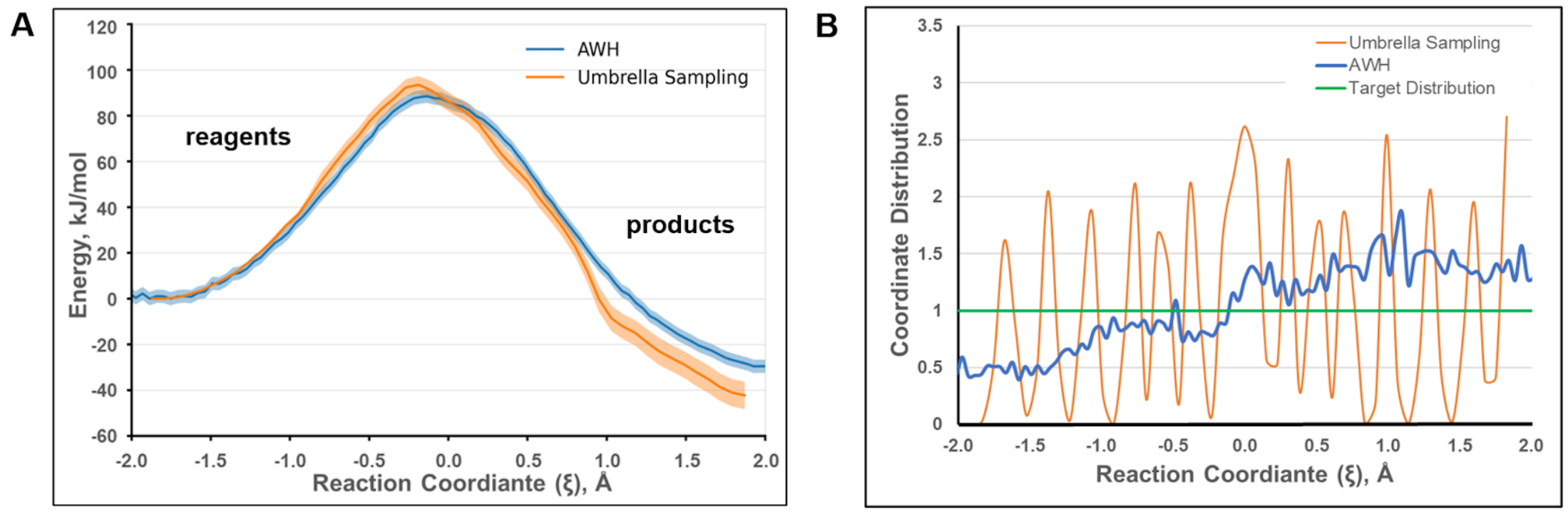

Figure S9: QM/MM free-energy simulations of ATP incorporation by SARS-CoV-2 RdRp. Panel A shows free-energy profiles obtained with US and multi-walker AWH. Standard error of US, evaluated with bootstrapping, and estimated target error of AWH is shown as a margins on the corresponding graph. Panel B displays sampled reaction-coordinate distributions. AWH drives the aggregate distribution closer to the target distribution and reaches a comparable barrier within 150 ps of aggregate QM/MM sampling compare to 640 ps in US case.

References

(1) Gamblin, T.; LeGendre, M.; Collette, M. R.; Lee, G. L.; Moody, A.; de Supinski, B. R.; Futral, S. The Spack package manager: bringing order to HPC software chaos. Proceedings of the International Conference for High Performance Computing, Networking, Storage and Analysis. New York, NY, USA, 2015.

(2) Building CP2K from source. Available at https://manual.cp2k.org/trunk/getting-started/build-from-source.html.

(3) Installation guide for GROMACS. Available at https://manual.gromacs.org/current/install-guide/index.html.

(4) Getting started with Spack. Available at https://spack.readthedocs.io/en/latest/getting_started.html.

(5) Gao, J.; Freindorf, M. Hybrid ab initio QM/MM simulation of N-methylacetamide in aqueous solution. *The Journal of Physical Chemistry A* **1997**, *101*, 3182–3188.

(6) Melo, M. C.; Bernardi, R. C.; Rudack, T.; Scheurer, M.; Riplinger, C.; Phillips, J. C.;

Maia, J. D.; Rocha, G. B.; Ribeiro, J. V.; Stone, J. E.; others NAMD goes quantum: an integrative suite for hybrid simulations. *Nature methods* **2018**, *15*, 351–354.

(7) Perdew, J. P.; Burke, K.; Ernzerhof, M. Generalized gradient approximation made simple. *Physical review letters* **1996**, *77*, 3865.

(8) VandeVondele, J.; Hutter, J. Gaussian basis sets for accurate calculations on molecular systems in gas and condensed phases. *The Journal of chemical physics* **2007**, *127*.

(9) Laino, T.; Mohamed, F.; Laio, A.; Parrinello, M. An efficient real space multigrid QM/MM electrostatic coupling. *Journal of Chemical Theory and Computation* **2005**, *1*, 1176–1184.

(10) Xu, J.; Deng, Q.; Chen, J.; Houk, K. N.; Bartek, J.; Hilvert, D.; Wilson, I. A. Evolution of shape complementarity and catalytic efficiency from a primordial antibody template. *Science* **1999**, *286*, 2345–2348.

(11) Lindahl, V.; Lidmar, J.; Hess, B. Accelerated weight histogram method for exploring free energy landscapes. *The Journal of chemical physics* **2014**, *141*.

(12) Aho, N.; Groenhof, G.; Buslaev, P. Do all paths lead to Rome? How reliable is umbrella sampling along a single path? *Journal of Chemical Theory and Computation* **2024**, *20*, 6674–6686.

(13) Bussi, G.; Donadio, D.; Parrinello, M. Canonical sampling through velocity rescaling. *J. Chem. Phys.* **2007**, *126*, 014101.

(14) Hess, B.; Bekker, H.; Berendsen, H. J. C.; Fraaije, J. G. E. M. LINCS: A linear constraint solver for molecular simulations. *J. Comput. Chem.* **1997**, *18*, 1463–1472.

(15) Hub, J. S.; De Groot, B. L.; Van Der Spoel, D. g_wham—A Free Weighted Histogram Analysis Implementation Including Robust Error and Autocorrelation Estimates. *Journal of chemical theory and computation* **2010**, *6*, 3713–3720.

(16) Morozov, D.; Modi, V.; Mironov, V.; Groenhof, G. The photocycle of bacteriophytochrome is initiated by counterclockwise chromophore isomerization. *The Journal of Physical Chemistry Letters* **2022**, *13*, 4538–4542.

(17) Grimme, S.; Antony, J.; Ehrlich, S.; Krieg, H. A consistent and accurate ab initio parametrization of density functional dispersion correction (DFT-D) for the 94 elements H-Pu. *The Journal of chemical physics* **2010**, *132*.

(18) Duan, Y.; Wu, C.; Chowdhury, S.; Lee, M. C.; Xiong, G. M.; Zhang, W.; Yang, R.; Cieplak, P.; Luo, R.; Lee, T.; Caldwell, J.; Wang, J. M.; Kollman, P. A point-charge force field for molecular mechanics simulations of proteins based on condensed-phase quantum mechanical calculations. *J. Comput. Chem.* **2003**, *24*, 1999–2012.

(19) Kirchdoerfer, R. N.; Ward, A. B. Structure of the SARS-CoV nsp12 polymerase bound to nsp7 and nsp8 co-factors. *Nature communications* **2019**, *10*, 2342.

(20) Gong, P.; Peersen, O. B. Structural basis for active site closure by the poliovirus RNA-dependent RNA polymerase. *Proceedings of the National Academy of Sciences* **2010**, *107*, 22505–22510.

(21) Webb, B.; Sali, A. Comparative protein structure modeling using MODELLER. *Current protocols in bioinformatics* **2016**, *54*, 5–6.

(22) Maier, J. A.; Martinez, C.; Kasavajhala, K.; Wickstrom, L.; Hauser, K. E.; Simmerling, C. ff14SB: improving the accuracy of protein side chain and backbone parameters from ff99SB. *Journal of chemical theory and computation* **2015**, *11*, 3696–3713.

(23) Jorgensen, W. L.; Chandrasekhar, J.; Madura, J. D.; Impey, R. W.; Klein, M. L. Comparison of simple potential functions for simulatin liquid water. *J. Chem. Phys.* **1983**, *79*, 926–935.
